# Comparative Genomic Profiles of *Salmonella* Typhimurium and *Salmonella* Dublin Bovine Isolates from the U.S. Indicate Possible Factors Associated with the Host Adaptation of *Salmonella* Dublin in the Region

**DOI:** 10.3390/microorganisms13040886

**Published:** 2025-04-12

**Authors:** Kingsley E. Bentum, Emmanuel Kuufire, Rejoice Nyarku, Viona Osei, Benjamin Adu-Addai, Jonathan G. Frye, Charlene R. Jackson, Temesgen Samuel, Woubit Abebe

**Affiliations:** 1 Center for Food Animal Health, Food Safety and Defense, Department of Pathobiology, College of Veterinary Medicine, Tuskegee University, Tuskegee, AL 36088, USA; kbentum8786@tuskegee.edu (K.E.B.); ekuufire9436@tuskegee.edu (E.K.); rnyarku8794@tuskegee.edu (R.N.); vosei3882@tuskegee.edu (V.O.); tsamuel@tuskegee.edu (T.S.); 2 Department of Biomedical Science, College of Veterinary Medicine, Tuskegee University, Tuskegee, AL 36088, USA; baduaddai@tuskegee.edu; 3 Poultry Microbiological Safety and Processing Research Unit, USDA-ARS, U.S. National Poultry Research Center, Athens, GA 30605, USA; jonathan.frye@usda.gov (J.G.F.); charlene.jackson@usda.gov (C.R.J.)

**Keywords:** *Salmonella* Dublin, *Salmonella* Typhimurium, serovar, antimicrobial resistance, cattle, host

## Abstract

*Salmonella* Dublin (*S.* Dublin) and *Salmonella* Typhimurium (*S.* Typhimurium) are commonly linked to bovine salmonellosis. *S.* Dublin is, however, considered a bovine-adapted serovar for primarily infecting and thriving in cattle. Using *S.* Typhimurium (a generalist serovar) as a benchmark, this study investigates genomic factors contributing to *S.* Dublin’s adaptation to cattle hosts in the U.S. A total of 1337 *S.* Dublin and 787 *S.* Typhimurium whole-genome sequences from bovine sources were analyzed with CARD (version 4.0.0), ARG-NOTT (version 6), and AMRfinderPlus (version 4.0.3) for antimicrobial resistance (AMR) genes; VFDB and AMRfinderPlus for virulence genes; AMRFinderPlus for stress genes; and Plasmidfinder for plasmids. Existing clonal groups among isolates of the two serovars were also investigated using the Hierarchical Clustering of Core Genome Multi-Locus Sequence Typing (HierCC-cgMLST) model. The results revealed minimal genomic variation among *S.* Dublin isolates. Comparatively, the IncX1 plasmid was somewhat exclusively identified in *S.* Dublin isolates and each carried an average of four plasmids (*p*-value < 0.05). Furthermore, *S*. Dublin isolates exhibited a higher prevalence of AMR genes against key antimicrobials, including aminoglycosides, beta-lactams, tetracyclines, and sulfonamides, commonly used in U.S. cattle production. Additionally, Type VI secretion system genes *tssJKLM* and *hcp2*/*tssD2*, essential for colonization, were found exclusively in *S.* Dublin isolates with over 50% of these isolates possessing genes that confer resistance to heavy metal stressors, like mercury. These findings suggest that *S.* Dublin’s adaptation to bovine hosts in the U.S. is supported by a conserved genetic makeup enriched with AMR genes, virulence factors, and stress-related genes, enabling it to colonize and persist in the bovine gut.

## 1. Introduction

*Salmonella* Dublin (*S*. Dublin) and *Salmonella* Typhimurium (*S*. Typhimurium) are among the common serovars associated with bovine salmonellosis [1]. Although the two commonly infect cattle, *S*. Dublin is described as a bovine-host-adapted serovar because it primarily infects and persistently thrives in the bovine host [2,3]. Calves are most susceptible to bovine salmonellosis, and infections caused by *S.* Dublin can result in systemic conditions such as meningoencephalitis, pneumonia, respiratory distress, hyperthermia, and diarrhea [2]. Similarly, those caused by *S.* Typhimurium can result in acute enteritis and exudative diarrhea [3].

A wide range of comparative genomic studies have been conducted to understand host–pathogen adaptation and the pathogenesis of *Salmonella* [4,5,6,7]. In the microbial world, an interplay of virulence factors, antimicrobial resistance genes, and stress response genes contributes to a pathogen’s ability to invade and thrive in its host [8,9]. For instance, to adapt to and survive in competitive host environments, pathogens utilize virulence genes to evade host defense systems and antimicrobial resistance genes to withstand antimicrobial therapies [10].

With the abundance of publicly available whole-genome sequence (WGS) data and analytical tools, it is now possible to conduct in-depth comparative analyses that provide insights beyond epidemiological evidence, extending to microbe-specific pathogenicity. One key advantage of such advanced technology is that a single next-generation sequencing (NGS) run can generate data on phylogenetics, antimicrobial resistance (AMR), and virulence profile of pathogens [11]. Furthermore, the sequence type (ST) of various *Salmonella* isolates can be obtained from their WGS data to aid in traceback investigations of human infections originating from food animals. For example, a recent study in China reported that *S*. Typhimurium ST34 from pigs and *S*. Typhimurium ST19 from chickens are the main serovars associated with gastro-infections in children and adults, respectively [12].

Given these benefits, major public health institutions in the U.S. now implement whole-genome sequencing of pathogens as part of routine investigations [13]. This has led to the creation of numerous pathogen sequence databases, widely accessible for research and investigation purposes [13]. Among the major public databases is the GenBank^®^ nucleic acid sequence database of the National Center for Biotechnology Information (NCBI), which contains approximately 3.7 billion nucleotide sequences from over 557,000 species [14,15]. Some countries have pathogen-specific databases, such as the open-access Chinese Local Salmonella Genome Database (CLSG), which provides essential surveillance information about *Salmonella* in the country [16]. These national databases provide data for other platforms such as EnteroBase [17].

Leveraging the wealth of publicly available genomic data, this study aimed to investigate the genomic factors that may contribute to the ability of the bovine-host-adapted serovar *S*. Dublin to thrive in its cattle host, using *S*. Typhimurium, a broad-host-range serovar, as a benchmark.

## 2. Materials and Methods

### 2.1. Data Acquisition

**Figure 1:**
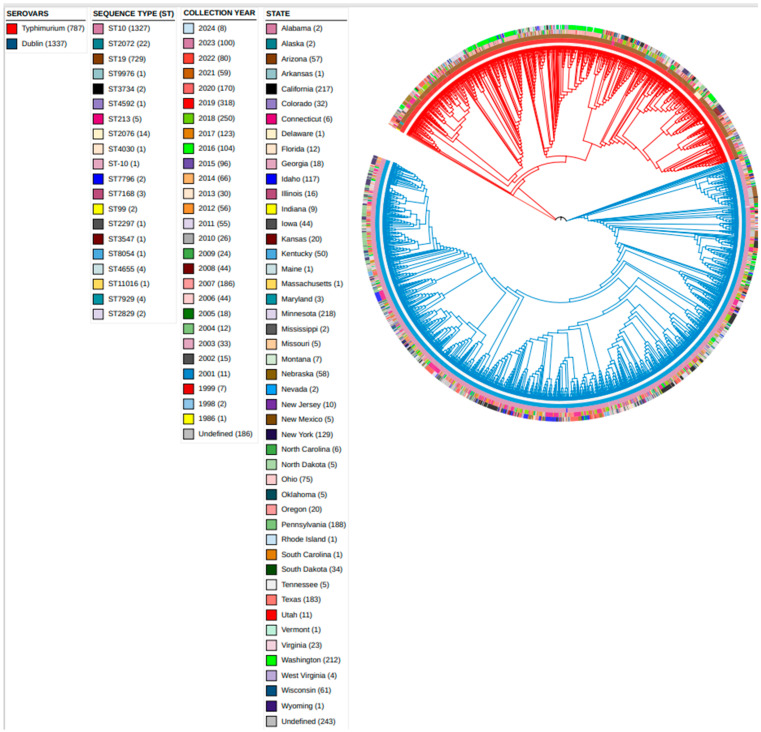
Phylogenetic tree displaying isolate metadata. The phylogenetic tree presents metadata for the isolates, including serovar, sequence type, collection year, and state of isolation. A legend for interpreting the tree is located on the left, with the number of isolates indicated in brackets. According to the legend order, the innermost track represents serovar designation, while the outermost track indicates the state of origin. *S.* Typhimurium isolates are shown with red branches, and *S.* Dublin isolates are shown with blue branches. The phylogenetic tree was plotted based on the NINJA Neighbor-Joining (NJ) algorithm in Enterobase.

All WGS data in this study were retrieved from Enterobase on 24 September 2024. At the time of accessing the Enterobase platform, there were a total of 523,825 curated *Salmonella* genomes available. Using the following search terms—serovar = Typhimurium/Dublin, country = United States, source niche = livestock, and source type = bovine—1370 *S*. Dublin- and 814 *S*. Typhimurium-assembled genomes were initially downloaded using a plug-in tab on the Enterobase platform. All genomic data available in Enterobase are automatically retrieved from Illumina short-read sequences available in various public short-read archives or those uploaded by registered users [17]. These short-read sequences are assembled using the automated and consistent QAssembly pipeline available in Enterobase. Genomes that pass quality control are finally genotyped [by Multilocus Sequence Typing (MLST), Ribosomal Multilocus Sequence Typing (rMLST), Core genome Multilocus Sequence Typing (cgMLST), and Whole genome Multilocus Sequence Typing (wgMLST)] and finally made accessible to the public together with their associated metadata [17]. The serovar definitions of the isolates were further validated with the Sistr program (https://github.com/phac-nml/sistr_cmd, accessed on 24 September 2024), and this reduced the data set to 1337 *S.* Dublin and 787 S. Typhimurium draft-assembled genomes, which can be accessed within the Enterobase database at (https://enterobase.warwick.ac.uk/species/senterica/search_strains?query=workspace:124148, accessed on 24 September 2024). The metadata of the isolates’ sequence type (ST), year of collection, and state of isolation are summarized in Figure 1. Details about the Enterobase QAssembly pipeline and genotyping methods can also be found at https://enterobase.readthedocs.io/en/latest/about.html, accessed on 25 September 2024.

### 2.2. Hierarchical Clustering of Core Genome Multilocus Sequence (HierCC-cgMLST) Analysis

Clonal groups of isolates were assigned using the Hierarchical Clustering of Core Genome Multi-Locus Sequence Typing (HierCC-cgMLST) plug-in within Enterobase, and this was accessed on 7 October 2024. For the HierCC-cgMLST, strains of serovars can differ up to a certain specified number of Core Genome Multilocus Sequence (cgMLST) alleles, which is indicated by the prefix “HC” followed by that number (e.g., HC5 will be for 5 cgMLST allelic differences) [18]. To investigate the clonal populations and the existing genetic diversities among the two serovars further, grape trees [19] were constructed using the cgMLST V2 + HierCC V1 scheme in Enterobase, which is based on a set of 3002 genes [20]. The NINJA Neighbor-Joining (NJ) algorithm [21] was employed in the construction of the grape tree for the 787 *S*. Typhimurium isolate genomes and 1337 *S*. Dublin isolate genomes.

### 2.3. Antimicrobial, Virulence, Stress Genes, and Plasmid Identification Analysis

The draft genome assemblies of the isolates were screened for AMR genes, virulence genes, stress genes, and plasmids using various databases. For AMR genes, three databases were used: the Comprehensive Antimicrobial Resistance Database (CARD) [22], AMRFinderPlus [23], and ARG-ANNOT [24]. For virulence genes, the VirulenceFinder [25] and the AMRFinderPlus databases were used. Stress genes were also obtained from the AMRFinderPlus results. Finally, PlasmidFinder [26] was used to identify plasmids within isolate sequences. Where more than one database was used, the independent results from the databases were aggregated to provide a single comprehensive result. Except for AMRFinderPlus (software version 4.0.3, database updated on version 2024-10-22.1), which was used in a standalone module, all other databases were updated on 24 November 2024, and used via ABRicate (version 1.0.1) (https://github.com/tseemann/abricate, accessed on 28 November 2024). For all ABRicate searches, the default Basic Local Alignment Search Tool (BLAST) threshold of 80% of minimum nucleotide identity and 80% coverage was used, as in similar studies [27].

### 2.4. Statistical Analysis

The statistical significance for the various comparative analyses was calculated with two-way ANOVA using GraphPad Prism (version 10.3.0). A *p*-value of <0.05 was deemed statistically significant.

## 3. Results

### 3.1. Grape Tree Phylogeny Based on HierCC-cgMLST

**Figure 2:**
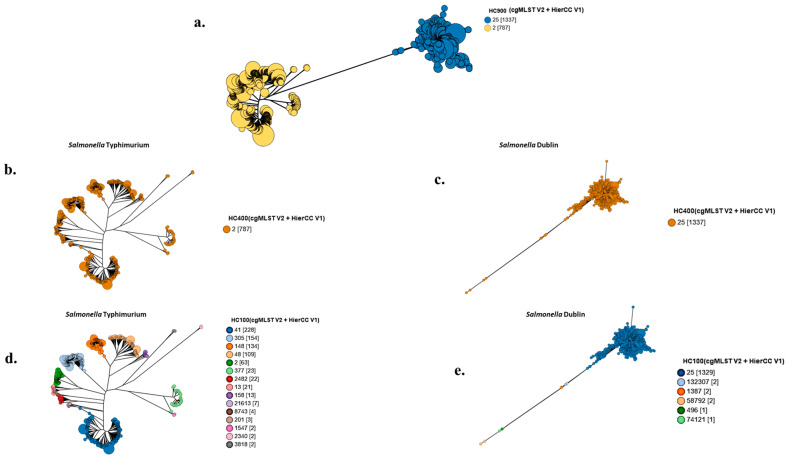
The GrapeTree of *Salmonella* Dublin and *Salmonella* Typhimurium isolates showing plots at the Hierarchical Clustering (HC) levels of HC900, HC400, and HC100. (**a**) A GrapeTree constructed at the HC900 level using the cgMLST V2 + HierCC V1 scheme based on a set of 3002 core genes and the Ninja Neighbor-Joining algorithm for a total of 2124 isolates comprising 787 *S*. Typhimurium and 1337 *S*. Dublin isolates, respectively. (**b**,**c**) Subtrees redrawn from (**a**) to show clustering results for *S*. Typhimurium (787 isolates) and *S*. Dublin (1337 isolates) at HC 400. (**d**,**e**) Subtrees redrawn from (**a**) to show clustering results for *S.* Typhimurium (787 isolates) and *S*. Dublin (1337 isolates) at HC100. All isolates are shown as colored nodes, and a figure legend for each plot is shown on the right side of the plot.

No variations were observed among isolates of the respective serovars at the Hierarchical Clustering levels 900 (HC900) and 400 (HC400) (Figure 2a–c). However, Hierarchical Clustering level 100 (HC100) showed that more variations existed among *S*. Typhimurium isolates than *S*. Dublin isolates. At this level, while 15 discrete clonal groups were observed among the *S*. Typhimurium isolates with the distribution of the isolates shared mainly among four major clusters, only 6 clonal groups were identified in *S*. Dublin isolates, with 99.4% (1329/1337) belonging to the HC100_25 cluster (Figure 2d,e).

### 3.2. Antimicrobial Resistance Genes

**Figure 3:**
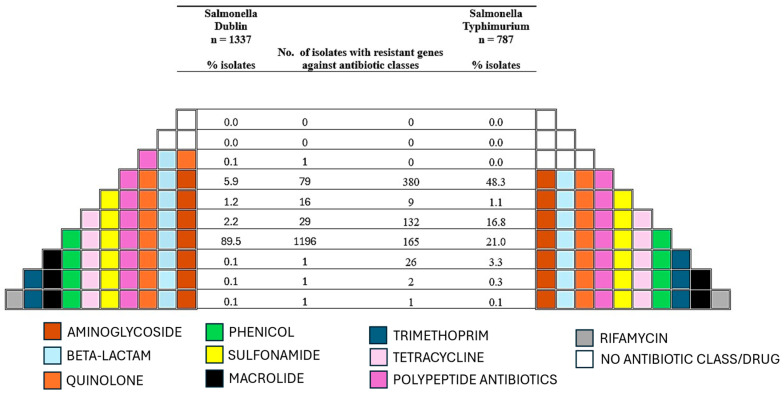
Antimicrobial resistance (AMR) genes against various drug classes identified among isolates of *Salmonella* Dublin and *Salmonella* Typhimurium. The colored pyramid shows the different drug classes for which AMR genes were detected in the various isolates. Each colored box represents a drug class as shown by the legend below the chart. The number of isolates within which the genes against the various antimicrobial drug classes were identified and their respective percentages are shown from center-left for *S.* Dublin and from center-right for *S*. Typhimurium (*p*-value < 0.05).

**Figure 4:**
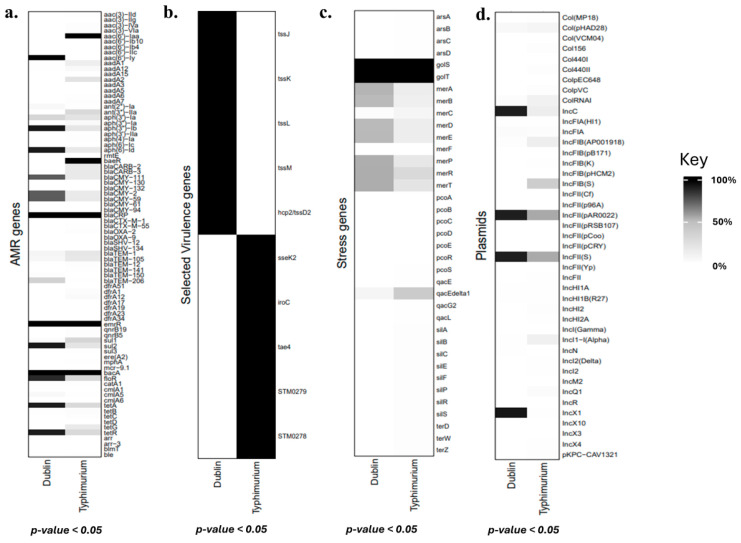
Heatmaps for genes and plasmids in the various isolates. (**a**) Antimicrobial genes, (**b**) selected virulence genes, (**c**) stress genes, and (**d**) plasmids identified in the isolates are shown in heatmaps. The scale for the heatmap is shown to the right, where black and white colors represent 100% and 0% of isolates of a given serovar, respectively.

Isolates of the two serovars mainly carried AMR genes against aminoglycosides, beta-lactam, tetracycline, sulfonamides, quinolone, phenicols, and polypeptide antibiotics (Figure 3). Out of these, 89.5% (1196/1337) of *S*. Dublin isolates carried AMR genes against aminoglycoside, beta-lactam, quinolone, sulfonamide, phenicol, tetracycline, and polypeptide antibiotics while 48.3% (380/787) of *S*. Typhimurium isolates carried AMR genes against aminoglycoside, beta-lactam, quinolone, and polypeptide antibiotics (*p*-value = 0.0001) (Figure 3). Among aminoglycoside resistance genes, *aac(6′)-Iy*, *aph(3″)-Ib*, and *aph(6)-Id* were present in over 90% of *S*. Dublin isolates. On the other hand, except for the *aac(6′)-Iaa* gene carried by all *S*. Typhimurium isolates, low proportions of isolates belonging to the serovar carried other resistance genes (Figure 4a). Among beta-lactam resistance genes, *baeR* was only found in *S*. Typhimurium isolates. All isolates from the two serovars, however, carried the *bla_CRP_* gene. Also, over 70% of *S*. Dublin isolates carried the genes *bla_CMY-111_*, *bla_CMY-2_*, and *bla_CMY-59_*, but they were found in less than 20% of *S*. Typhimurium isolates (Figure 4a). For tetracycline resistance, the *tetA* and *tetR* genes were found in more than 90% of *S*. Dublin isolates but in less than 30% of *S*. Typhimurium isolates. For sulfonamide resistance, *sul2* was the prevalent gene in *S*. Dublin found in 92.3% (1234/1337) of isolates. However, less than 30% of *S*. Typhimurium isolates carried either the *sul1* or *sul2* gene (Figure 4a). For resistance against quinolone and polypeptide antibiotics, all isolates from the two serovars carried the *emrR* and *bacA* genes, respectively (Figure 4a). The main gene for phenicol resistance was *floR,* which was found in 88% (1176/1337) and 31.1% (245/787) of *S*. Dublin and *S*. Typhimurium isolates, respectively. All AMR genes detected among the isolates in this study are presented in the Supplementary Data (AMR genes). Finally, almost 100% of isolates of the two serovars were equipped with various efflux and multidrug-resistance-related genes, which are also shown in Supplementary Data (Multidrug and Efflux genes).

### 3.3. Virulence Genes

**Figure 5:**
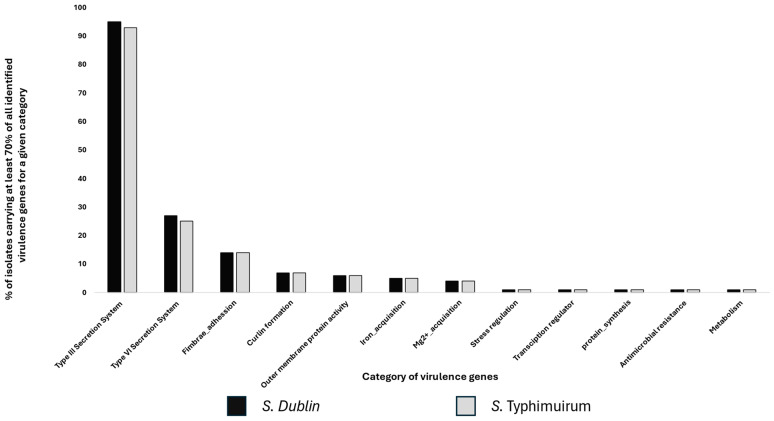
Bar chart showing the percentage of isolates with selected virulence genes. The bar chart displays the percentage of isolates carrying at least 70% of all identified genes within each functional category of virulence genes. The y-axis indicates the percentage of isolates, while the x-axis represents the different functional categories of virulence genes. Black bars represent *S*. Dublin isolates, and gray bars represent *S*. Typhimurium isolates. The legend is provided at the bottom of the figure.

The majority (70–100%) of isolates from both serovars carried genes with functions related to the Type III secretion system (T3SS), fimbriae adhesion, Type VI secretion system (T6SS), curlin formation, outer membrane protein regulatory activities, stress regulation, and iron and magnesium acquisition. However, *S*. Dublin isolates comparatively carried more genes related to T3SS and T6SS (Figure 5). The genes *tssJ*, *tssK*, *tssL*, *tssM*, and *hcp2*/*tssD2*, all related to the T6SS, were found in almost 100% of *S*. Dublin isolates but were entirely missing in all *S.* Typhimurium isolates (Figure 4b). On the other hand, the T6SS gene *tae4* and gene clusters STM0278 and STM0279, and the T3SS genes *ssek2* and *iroC* were found in over 90% of the *S*. Typhimurium isolates but were scarcely detected in the *S*. Dublin isolates (Figure 4b). All virulence-associated genes detected among the isolates in this study are presented in the Supplementary Data (Virulence genes).

### 3.4. Stress Genes

Thirty-seven (37) stress genes were identified from isolates of the two serovars, with the *golT* and *golS* genes identified in over 90% of both *S*. Dublin and *S*. Typhimurium isolates (Figure 4c). Nevertheless, more than 50% of *S.* Dublin isolates carried the genes *merA*, *merB*, *merD*, *merE*, *merP, merR,* and *merT,* which were found in less than 30% of *S.* Typhimurium isolates (Figure 4c).

### 3.5. Plasmids

**Figure 6:**
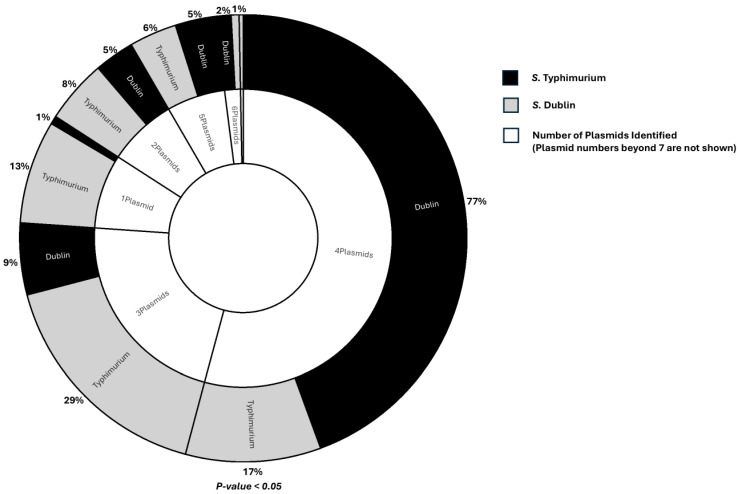
The chart illustrates the proportion of *Salmonella* Dublin and *Salmonella* Typhimurium isolates carrying at least one plasmid. The percentage of isolates from each serovar, based on the number of plasmids they carry, is indicated within the corresponding segments on the outer part of the chart. Black and gray shading represent *S.* Dublin and *S.* Typhimurium, respectively, as indicated in the legend to the right of the chart.

More than 50% of the isolates from both serovars carried the plasmids IncFII(pAR0022) and IncFII(S). Additionally, over 90% of *S.* Dublin isolates harbored both the IncC and IncX1 plasmids. In contrast, among the *S.* Typhimurium isolates, the IncC plasmid was present at low levels, while the IncX1 plasmid was completely absent (Figure 4d). More plasmids were carried by *S.* Dublin isolates than *S.* Typhimurium isolates (Figure 6).

## 4. Discussion

This study highlights the value of utilizing publicly available genomic data for research purposes. It shows that *S.* Dublin possesses a conserved genomic “toolbox” that includes virulence genes for colonization, stress response genes against heavy metals commonly found in soil, and antimicrobial resistance genes targeting antibiotics frequently used in cattle production.

To begin, this study has demonstrated that the genome of the bovine-host-adapted serovar *S.* Dublin is highly conserved and exhibits little diversity compared to *S.* Typhimurium, which is described as a generalist serovar due to its broad host range [28]. The conserved genome of *S*. Dublin has been attributed to its unique adaptation as a bovine-host-adapted serovar [29]. On the other hand, the multiple clustering of *S*. Typhimurium at HC100, as also observed in other studies [30], confirms its polyphyletic nature [20] and has also been linked to its generalist characteristics [31].

According to the 2020 summary report from the U.S. Food and Drug Administration (FDA), 41% of domestic sales and the distribution of medically important antimicrobials approved for use in food-producing animals were intended for cattle [32]. This report also showed that, among these sales, 80% of cephalosporins, 57% of sulfonamides, 54% of aminoglycosides, 43% of tetracyclines, and 11% of penicillins were designated for cattle use [32]. This study highlights the multidrug-resistant (MDR) nature of *S.* Dublin isolates from bovine sources in the U.S., showing that, on average, these isolates carry resistance genes against aminoglycosides, beta-lactams, quinolones, sulfonamides, phenicols, tetracyclines, and polypeptide antibiotics (specifically bacitracin). Except for phenicols (specifically chloramphenicol), which have long been banned in the U.S. and many other countries for use in food-producing animals [33,34], all the aforementioned drugs are still used. However, bacitracin is not classified as medically important by the FDA, though it continues to be used due to claims of its growth-promoting effects in cattle [35]. Interestingly, this aligns with our finding that the *bacA* gene, which confers resistance to bacitracin [36], was among the few genes consistently present in all *S*. Dublin and *S*. Typhimurium isolates.

Further analysis showed that *S.* Dublin appears better equipped with antimicrobial resistance (AMR) genes to counteract drugs approved for use in U.S. cattle production. For instance, over 90% of *S*. Dublin isolates carried the genes *aac(6′)-Iy*, *aph(3″)-Ib*, and *aph(6)-Id*, which are associated with aminoglycoside resistance [37]. While *S*. Typhimurium isolates also carried AMR genes against aminoglycosides, their prevalence is comparatively lower, with none exceeding 30%, except for *aac(6′)-Iaa*, which is a cryptic non-functional gene [38]. Regarding cephalosporins, a subclass of beta-lactam antibiotics [39], the *bla_CRP_* gene was present in all isolates of both serovars. However, an interesting observation deserving further investigation was the exclusive presence of the *baeR* gene in *S*. Typhimurium isolates, which plays a role in the susceptibility of microbes to cephalosporin [40]. Over 70% of *S*. Dublin isolates carried the *bla_CMY-111_*, *bla_CMY-2_*, and *bla_CMY-59_* genes, whereas they were found in less than 30% of *S*. Typhimurium isolates. This suggests that *S*. Dublin may have a greater capacity to resist cephalosporin antibiotics. Similar trends were observed with AMR genes against tetracyclines and sulfonamides. For sulfonamide resistance, the *sul2* gene was the most prevalent among *S*. Dublin isolates and may be the preferred gene for sulfonamide resistance in *S.* Dublin. Although both the *sul1* and the *sul2* genes are reported to have the same frequency of detection among sulfonamide-resistant Gram-negative microbes [41], the *sul2* gene was the most dominant gene among most sulfonamide-resistant *Salmonella* spp. in a study by Pavelquesi et al. [42]. A large proportion (over 90%) of *S.* Dublin isolates also carried genes against tetracycline resistance, which was not the case for *S.* Typhimurium isolates in this study. The abundance of these AMR genes in *S.* Dublin suggests that this serovar is more likely to resist treatment and prolong infections in its bovine host. All isolates from both serovars carried the *emR* gene, which confers resistance to quinolone drugs. This may be attributed to the widespread and indiscriminate use of these drugs in food animal production. However, the presence of antimicrobial resistance (AMR) genes does not necessarily indicate phenotypic resistance [43]. Further studies are therefore needed to investigate the antimicrobial susceptibilities of these isolates.

The origins of the AMR genes identified in these isolates were not investigated, due to computational limitations. However, the significantly higher number of AMR genes in *S.* Dublin isolates compared to *S*. Typhimurium could be attributed to plasmids, as these mobile genetic elements play a crucial role in the acquisition and transfer of AMR genes [44,45]. Most isolates of both serovars carried the IncFII-type and IncC plasmids, which are well known to be associated with MDR [46,47,48,49,50]. However, in addition to the fact that *S*. Dublin isolates carried more of these plasmids than *S*. Typhimurium isolates on average, a noteworthy observation was the presence of the IncX1 plasmid in over 90% of *S.* Dublin isolates, which were virtually missing among *S.* Typhimurium isolates. This interesting finding suggests that the plasmid may be important in the biology of *S.* Dublin. This IncX1 plasmid is noted for carrying MDR genes [51]. In addition, a study in China identified arsenic-resistant operons on this plasmid [52]. Although arsenic-resistant genes were not identified in the *S*. Dublin isolates in this study, over 50% carried genes of the mer operon, notable for mercury resistance [53]. *S*. Dublin isolates carrying mercury-resistant genes coupled with the association of the IncX1 plasmid with MDR and arsenic resistance may be a special adaptation to thrive in the gut of its host, as the grazing habit of cattle makes them prone to ingesting heavy metals such as mercury, which may be contained in soil [54]. Although this study reports the presence of these plasmids, further studies are needed to determine their functional competencies.

Almost all isolates from both serovars presented a similar profile for virulence genes with activities relating to T3SS, T6SS, magnesium and iron acquisition, curlin formation, and outer-membrane proteins. These are essential and core to *Salmonella* virulence [55,56,57,58,59]. However, among the subtle but significant observations in this study was that *S*. Dublin isolates had over 70% of the genes related to T3SS and T6SS compared to *S*. Typhimurium. More specifically, the *tssJKLM* genes, which are key components of the baseplate and membrane complex of T6SS [60], and the *tssD-2*/*hcp2* gene, also essential for colonization [61], were found only among *S*. Dublin isolates. Generally, T6SS is instrumental in conferring a competitive advantage against other microbiota [62]. The presence of these genes in *S.* Dublin may help the serovar outcompete other microbes in colonizing the gut of its bovine host. Conversely, the *sseK2* and *sspH2* genes, key factors in host immune evasion [63,64], were predominantly found in *S*. Typhimurium and nearly absent in *S.* Dublin isolates. Similarly, the hcp2 gene clusters STM0278 and STM0279, located in the mid-region of the T6SS, were also missing in *S*. Dublin [65]. This absence warrants further investigation, as a study in Germany also reported that the *sspH2* gene was missing in 78 cattle-derived *S*. Dublin isolates [66]. Additionally, while both *iroB* and *iroC* genes of the *iro* operon are important for iron uptake and oxidative stress defense [67], both were found in nearly all *S*. Typhimurium isolates. Only *iroB* was, however, detected in *S*. Dublin, a phenomenon that also deserves further exploration [68].

Although this study incorporated multiple databases to increase the stringency and comprehensiveness of our results, its findings are limited by the genes populating the databases used at the time of our analysis. Continued advancements in bioinformatics and the growing availability of whole-genome sequences will enable deeper investigations into *Salmonella* serovars’ pathogenicity and host adaptations in the future.

## 5. Conclusions

This study reveals that *S.* Dublin isolates in the U.S. have a highly conserved genomic profile, equipped with a repertoire of AMR genes against the antimicrobials commonly used in cattle production. The presence of virulence and stress-tolerance genes also enhances *S*. Dublin’s ability to colonize and persist within the bovine gut, contributing to its stability and persistence in cattle and posing challenges for treatment and control.

## Data Availability

All whole-genome sequences of isolates used for this study can be located within the Enterobase database using this link: https://enterobase.warwick.ac.uk/species/senterica/search_strains?query=workspace:124148 (accessed on 24 September 2024).

## Supplementary Materials

The following supporting information can be downloaded at: https://www.mdpi.com/article/10.3390/microorganisms13040886/s1, Supplementary Data.
